# ASSOCIATION BETWEEN PATTERNS OF SOCIAL ACTIVITIES AND FRAILTY STATUS AMONG OLDER ADULTS IN CHINA

**DOI:** 10.1093/geroni/igae098.3225

**Published:** 2024-12-31

**Authors:** Sok Leng Che, Ka Kei Chao, Ka Kit Wong

**Affiliations:** Kiang Wu Nursing College of Macau, Macao, Macao, Macau; Kiang Wu Nursing College of Macau, Macao, Macao, Macau; Kiang Wu Nursing College of Macau, Macao, Macao, Macau

## Abstract

Loneliness has been identified as a public health priority by WHO. Studies have indicated that participating in social activities could reduce loneliness and the probability of being frail. This study examines social activity among Chinese older adults and its impact on frailty. This investigation utilizes data from 10,762 participants aged 60 years and over in the 2018 wave of the China Health and Retirement Longitudinal Survey (CHARLS). Frailty status was assessed by constructing a Frailty Index using health deficit accumulation model. Latent class analysis was employed to explore patterns of social activities, and logistic regression was used to identify factors associated with frailty status. The study identified five distinct types of social activity pattern, e.g. face-to-face interaction, socially active and diverse, socially inactive, socializing through the role of caregiver, and socializing through the Internet. Older adults in China are characterized by being socially inactive, accounting for 82% (n=8,827) of the total participants. Compared to older adults who practice face-to-face interaction, older adults who are identified as socially inactive (aOR=2.05, p< 0.001, 95% C.I.=1.68 to 2.51), and socialize in the role of caregiver (aOR=1.43, p=0.01, 95% C.I.=1.08 to 1.88) showed significantly higher odds of being frail. Older adults who hold the role of caregiver are particularly susceptible to frailty. The caregiving role may restrict their social network, while social inactivity may increase the risk of social isolation. Therefore, older individuals exhibiting these patterns of social activity may benefit from enhanced community and family support.

